# The melatonin receptor genes are linked and associated with the risk of polycystic ovary syndrome

**DOI:** 10.1186/s13048-024-01343-1

**Published:** 2024-01-13

**Authors:** Teodor T. Postolache, Qamar M. Al Tinawi, Claudia Gragnoli

**Affiliations:** 1 Mood and Anxiety Program, Department of Psychiatry, University of Maryland School of Medicine, Baltimore, MD 21201 USA; 2 Rocky Mountain Mental Illness Research Education and Clinical Center (MIRECC), Consortium for Research and Education (MVM-CoRE), Denver, CO 80246 USA; 3 Mental Illness Research Education and Clinical Center (MIRECC), Veterans Integrated Service Network (VISN) 5, VA Capitol Health Care Network, Baltimore, MD 21090 USA; 4 Department of Medicine, Creighton University School of Medicine, Omaha, NE 68124 USA; 5 Division of Endocrinology, Department of Medicine, Creighton University School of Medicine, Omaha, NE 68124 USA; 6 Department of Public Health Sciences, Penn State College of Medicine, Hershey, PA 17033 USA; 7 Molecular Biology Laboratory, Bios Biotech Multi-Diagnostic Health Center, Rome, 00197 Italy

**Keywords:** Polycystic ovarian syndrome, PCOS, Melatonin, Melatonin receptor, Melatonin receptor 1B, *MTNR1B*, Melatonin receptor 1A, *MTNR1A*, Gene, Variant, Linkage disequilibrium, Association, Ovary

## Abstract

Polycystic ovarian syndrome (PCOS) is a genetically complex disorder that involves the interplay of multiple genes and environmental factors. It is characterized by anovulation and irregular menses and is associated with type 2 diabetes. Neuroendocrine pathways and ovarian and adrenal dysfunctions are possibly implicated in the disorder pathogenesis. The melatonin system plays a role in PCOS. Melatonin receptors are expressed on the surface of ovarian granulosa cells, and variations in the melatonin receptor genes have been associated with increased risk of PCOS in both familial and sporadic cases. We have recently reported the association of variants in *MTNR1A* and *MTNR1B* genes with familial type 2 diabetes. In this study, we aimed to investigate whether *MTNR1A* and *MTNR1B* contribute to PCOS risk in peninsular families. In 212 Italian families phenotyped for PCOS, we amplified by microarray 14 variants in the *MTNR1A* gene and 6 variants in the *MTNR1B* gene and tested them for linkage and linkage disequilibrium with PCOS. We detected 4 variants in the *MTNR1A* gene and 2 variants in the *MTNR1B* gene significantly linked and/or in linkage disequilibrium with the risk of PCOS (*P* < 0.05). All variants are novel and have not been reported before with PCOS or any of its related phenotypes, except for 3 variants previously reported by us to confer risk for type 2 diabetes and 1 variant for type 2 diabetes-depression comorbidity. These findings implicate novel melatonin receptor genes’ variants in the risk of PCOS with potential functional roles.

## Background

Melatonin is a pineal hormone known for its role in the regulation of circadian [1] and seasonal rhythms [2], in addition to glucose and lipid metabolism [3, 4] with a role in obesity [5], anti-inflammation and antioxidation [6–8].

Melatonin exerts its roles through two melatonin G protein-coupled receptors: melatonin receptor 1 A (encoded by *MTNR1A* gene) and melatonin receptor 1B (encoded by *MTNR1B* gene) [9, 10]. The two receptors are expressed on the nervous system, pancreas, liver, skeletal muscle, adipose tissue, and ovaries [6, 11]. The two melatonin receptors have been implicated in the risk of mental and metabolic disorders such as type 2 diabetes (T2D) [12–14] and depression (MDD) [14, 15].

Of interest, polycystic ovarian syndrome (PCOS), a complex and common hormonal disorder affecting women of reproductive age and characterized by anovulation, hyperandrogenism, and polycystic ovaries, is commonly associated with type 2 diabetes (T2D) [16] and mental traits, including anxiety [17, 18] and depression [19, 20]. PCOS, which affects approximately 6-18% of women worldwide [21] and can lead to long-term health consequences, including infertility, metabolic syndrome, cardiovascular disease, is a genetically complex disorder that involves the interplay of multiple genes and environmental factors [22]. PCOS is linked to a variety of possible pathogenetic impairments, distinct or overlapping, including the neuroendocrine pathways, and ovarian and adrenal hormonal secretions [23]. The circadian rhythm and melatonin system have been implicated in PCOS [24]. Candidate gene studies have associated PCOS with several genes, including the insulin receptor gene (*INSR*) [25], insulin-like growth factor (IGF) system genes, luteinizing hormone (LH) /chorionic gonadotropin receptor gene (*LHCGR*) [26], genes involved in androgen biosynthesis and steroid hormone metabolism (*CYP11*) [25], and the melatonin receptor genes (*MTNR1A* and *MTNR1B*) [27]. Melatonin receptors are expressed on the surface of ovarian granulosa cells [28] and variations in the melatonin receptor genes have been associated with increased risk of PCOS in both familial and sporadic cases [27]. We have recently reported the linkage and linkage plus association of variants in *MTNR1A* with familial type 2 diabetes [13] and in *MTNR1B* [14] with familial type 2 diabetes and type 2 diabetes-depression comorbidity. In this study, we aimed to investigate whether the *MTNR1A* and *MTNR1B* genes are in linkage to and/or linkage disequilibrium (LD, i.e., association joint to linkage) with PCOS in Italian families.

## Materials and methods

We originally recruited for a type 2 diabetes (T2D) study 212 Italian families, which were later phenotyped for PCOS according to the phenotypes for PCOS necessary to meet the Rotterdam diagnostic criteria (presence of at least two of these three characteristics: chronic anovulation or oligomenorrhea, clinical or biochemical hyperandrogenism, and/or polycystic ovaries) [29]. We amplified by microarray 14 variants in the *MTNR1A* gene and 6 variants in the *MTNR1B* gene and tested them for linkage and LD with PCOS, using Pseudomarker [30] with dominant and recessive models with complete or incomplete penetrance, after excluding genotyping and Mendelian errors via PLINK [31]. The first tool (Pseudomarker) offers a robust method to simultaneously examine linkage and LD in a combination of family and singleton samples, utilizing the true pedigree relationships without relying on artificial assumptions to rectify linkage effects in statistics [30]. The second tool (PLINK) is a well-known toolset for whole genome association analysis that is both free and open-source, engineered to efficiently conduct various fundamental, large-scale analyses. We used the correlation coefficient of variants with data from the 1000 Genomes Project (https://www.internationalgenome.org/data-portal/population/TSI) to estimate the presence of LD blocks.

*In-Silico* Analysis. We used different bioinformatics tools to predict the risk variants’ roles in transcription factor (TF) binding (SNP Function Prediction) [32], miRNA binding (mirSNP) [33], splicing (SpliceAI) [34], and regulatory potential (RegulomeDB) [35].

## Results and discussion

**Fig. 1:**
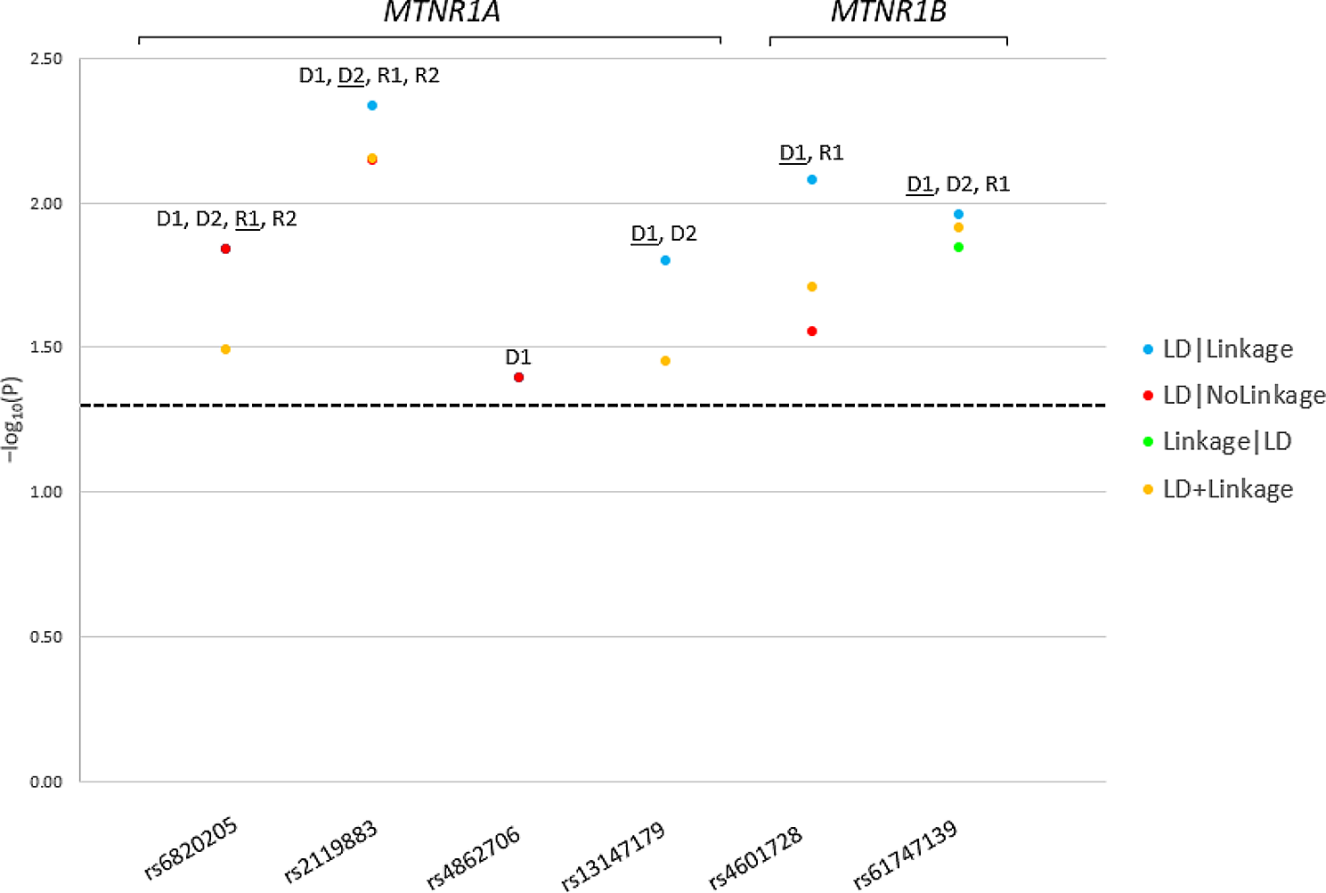
Parametric Analysis Results of Polycystic Ovarian Syndrome (PCOS) *MTNR1A*- and *MTNR1B*-Risk Single Nucleotide Polymorphisms (SNPs)

We detected 4 variants in the *MTNR1A* gene and 2 variants in the *MTNR1B* gene significantly linked and/or associated (LD) with the risk of PCOS (*P* < 0.05) (Table 1). The variants were significant across different inheritance models (Fig. 1). Two variants (rs2119883 and rs13147179) were within an LD block (Set01). All variants are novel and have not been reported before with PCOS or any of its related phenotypes (i.e., T2D, obesity, insulin resistance, metabolic syndrome, hyperglycemia, oligomenorrhea, anovulation, irregular menses, hyperandrogenism, MDD, male-pattern baldness, acne, hirsutism, infertility), excluding T2D for 3 risk variants and T2D-MDD for 1 risk variant. Within peninsular families, the same risk alleles of the two variants (*MTNR1B*-rs61747139 and *MTNR1A*-rs2119883) were previously linked to and associated with the risk of T2D [13, 14], confirming the interrelatedness of these complex phenotypes. On the other hand, the non-risk alleles of the two variants (*MTNR1A*-rs13147179 and *MTNR1B*-rs4601728) were linked and associated with T2D and T2D-MDD comorbidity respectively [13, 14], indicating multiple association at the allelic level and possibly the presence of LD with other contributing yet undetected variants. Via our bioinformatic analyses, we found that the risk allele (A) of the variant *MTNR1A*-rs13147179 disrupts the binding of Kruppel-like factor 5 (KLF5) which is hypomethylated in the ovarian tissue in PCOS [36], potentially extending the role of this variant to epigenetic mechanisms. We also found that the risk allele (G) of the variant *MTNR1B*-rs61747139 affects the binding of transcription factor AP2A (TFAP2A) which is expressed in the brain, liver, pancreas, and ovaries [37] and forms a part of the signaling network of PCOS at least in vitro [38]. PCOS patients in our study could therefore be at higher risk due to altered expression of genes in PCOS pathways. Our study therefore implicates novel melatonin receptor genes’ variants in the risk of PCOS with potential functional roles. It also offers the possibility of inhibitors of melatonin metabolism (e.g., coumarins [39]) as novel therapeutic modalities in the treatment of PCOS. However, more studies are needed to validate these results.

**Table 1:** Polycystic Ovarian Syndrome (PCOS) *MTNR1A*- and *MTNR1B*-Risk Single Nucleotide Polymorphisms (SNPs)

Gene	Model^1^	SNP	Position	Ref	Alt	Risk Allele	Consequence	LD block	Reported in PCOS or related phenotype?^2^
*MTNR1A*	D1, D2, R1, R2	rs6820205	186,543,713	T	C	C	Intronic	Independent	Novel
	D1, D2, R1, R2	rs2119883	186,547,921	C	T	T	Intronic	Set01	T2D^3^ [13]
	D1	rs4862706	186,552,540	G	A	A	Intronic	Independent	Novel
	D1, D2	rs13147179	186,554,365	G	A	A	Intronic	Set01	T2D^3^ [13]
*MTNR1B*	D1, R1	rs4601728	92,971,992	A	G	G	Intronic	Independent	T2D-MDD^4^ [14]
	D1, D2, R1	rs61747139	92,981,951	A	G	G	Missense	Independent	T2D^3^ [14]

^1^Models: D1: dominant, complete penetrance, D2: dominant, incomplete penetrance, R1: recessive, complete penetrance, R2: recessive, incomplete penetrance. ^2^(i.e., type 2 diabetes, obesity, insulin resistance, metabolic syndrome, hyperglycemia, oligoamenorrhea, anovulation, irregular menses, hyperandrogenism, male-pattern baldness, acne, hirsutism, infertility). ^3^T2D=type 2 diabetes, ^4^MDD=major depressive disorder)

## Data Availability

The study data are available on reasonable request, and due to lacking specific patients’ consent and privacy restrictions, they are not publicly available.

## Declarations

### Authors’ information

C.G. is Professor of Medicine and Chief of Endocrinology and Endowed Puller Chair at Creighton University School of Medicine, Omaha, NE, and Adjunct Professor of Public Health Sciences, Penn State University College of Medicine, Hershey, PA; Q.M.A.T. is an internal medicine resident at Creighton University School of Medicine, Omaha, NE. T.T.P. is a Professor of Psychiatry at the University of Maryland School of Medicine, Baltimore, MD, and a research investigator with the Rocky Mountain MIRECC, Aurora, CO, and the VISN 5 MIRECC, Baltimore, MD.

## References

[1] Lewy AJ (1992). Melatonin shifts human circadian rhythms according to a phase-response curve. Chronobiol Int.

[2] Bartness TJ (1993). The timed infusion paradigm for melatonin delivery: what has it taught us about the melatonin signal, its reception, and the photoperiodic control of seasonal responses?. J Pineal Res.

[3] Genario R (2021). Melatonin supplementation in the management of obesity and obesity-associated disorders: a review of physiological mechanisms and clinical applications. Pharmacol Res.

[4] Garaulet M (2020). Melatonin effects on glucose metabolism: Time to unlock the controversy. Trends Endocrinol Metab.

[5] Guan Q et al. *Mechanisms of melatonin in obesity: a review*. Int J Mol Sci, 2021. 23(1). 10.3390/ijms23010218 PMC8745381 35008644

[6] Pandi-Perumal SR (2008). Physiological effects of melatonin: role of melatonin receptors and signal transduction pathways. Prog Neurobiol.

[7] Ferlazzo N et al. *Is melatonin the cornucopia of the 21st Century?*. Antioxid (Basel), 2020. 9(11). 10.3390/antiox9111088 PMC7694322 33167396

[8] Chitimus DM et al. *Melatonin’s Impact on Antioxidative and Anti-Inflammatory Reprogramming in Homeostasis and Disease*. Biomolecules, 2020. 10(9). 10.3390/biom10091211 PMC7563541 32825327

[9] Li DY (2013). Melatonin receptor genes in vertebrates. Int J Mol Sci.

[10] Emet M (2016). A review of Melatonin, its receptors and drugs. Eurasian J Med.

[11] Slominski RM (2012). Melatonin membrane receptors in peripheral tissues: distribution and functions. Mol Cell Endocrinol.

[12] Karamitri A, Jockers R (2019). Melatonin in type 2 diabetes mellitus and obesity. Nat Rev Endocrinol.

[13] Amin M, Gragnoli C. Melatonin receptor 1A gene (MTNR1A) linkage and association to type 2 diabetes in Italian families. Eur Rev Med Pharm Sci (In Press; 2023. 10.26355/eurrev_202305_32480 37259752

[14] Amin M et al. *The role of melatonin receptor 1B gene (MTNR1B) in the susceptibility to depression and type 2 diabetes comorbidity* Genes & Diseases (In Press), 2023. 10.1016/j.gendis.2023.06.036 PMC10825273 38292205

[15] Galecka E (2011). Single nucleotide polymorphisms and mRNA expression for melatonin MT(2) receptor in depression. Psychiatry Res.

[16] Diamanti-Kandarakis E, Dunaif A (2012). Insulin resistance and the polycystic ovary syndrome revisited: an update on mechanisms and implications. Endocr Rev.

[17] Deeks AA, Gibson-Helm ME, Teede HJ (2010). Anxiety and depression in polycystic ovary syndrome: a comprehensive investigation. Fertil Steril.

[18] Wang Y, Ni Z, Li K (2021). The prevalence of anxiety and depression of different severity in women with polycystic ovary syndrome: a meta-analysis. Gynecol Endocrinol.

[19] Kolhe JV (2022). PCOS and depression: common links and potential targets. Reprod Sci.

[20] Xing L (2022). Depression in polycystic ovary syndrome: focusing on pathogenesis and treatment. Front Psychiatry.

[21] Joham AE (2022). Polycystic ovary syndrome. Lancet Diabetes Endocrinol.

[22] Unluturk U (2007). The genetic basis of the polycystic ovary syndrome: a Literature Review including discussion of PPAR-gamma. PPAR Res.

[23] Rosenfield RL, Ehrmann DA (2016). The pathogenesis of polycystic ovary syndrome (PCOS): the hypothesis of PCOS as functional ovarian hyperandrogenism revisited. Endocr Rev.

[24] Wang F (2021). Association between circadian rhythm disruption and polycystic ovary syndrome. Fertil Steril.

[25] Seyed Abutorabi E (2021). Investigation of the FSHR, CYP11, and INSR mutations and polymorphisms in Iranian infertile women with polycystic ovary syndrome (PCOS). Rep Biochem Mol Biol.

[26] Singh S (2022). Association analysis of LHCGR variants and polycystic ovary syndrome in Punjab: a case-control approach. BMC Endocr Disord.

[27] Yi S et al. *Association between melatonin receptor gene polymorphisms and polycystic ovarian syndrome: a systematic review and meta-analysis*. Biosci Rep, 2020. 40(6). 10.1042/BSR20200824 PMC7317604 32463080

[28] Woo MM (2001). Direct action of melatonin in human granulosa-luteal cells. J Clin Endocrinol Metab.

[29] Rotterdam EA (2004). .-S.P.c.w.g., *revised 2003 consensus on diagnostic criteria and long-term health risks related to polycystic ovary syndrome (PCOS)*. Hum Reprod.

[30] Hiekkalinna T (2011). PSEUDOMARKER: a powerful program for joint linkage and/or linkage disequilibrium analysis on mixtures of singletons and related individuals. Hum Hered.

[31] Purcell S (2007). PLINK: a tool set for whole-genome association and population-based linkage analyses. Am J Hum Genet.

[32] Xu Z, Taylor JA. *SNPinfo: Integrating GWAS and candidate gene information into functional SNP selection for genetic association studies* Nucleic Acids Research, 2009. 37(SUPPL. 2). 10.1093/nar/gkp290 PMC2703930 19417063

[33] Liu C (2012). MirSNP, a database of polymorphisms altering miRNA target sites, identifies miRNA-related SNPs in GWAS SNPs and eQTLs. BMC Genomics.

[34] Jaganathan K (2019). Predicting Splicing from primary sequence with deep learning. Cell.

[35] Boyle AP (2012). Annotation of functional variation in personal genomes using RegulomeDB. Genome Res.

[36] Cao P (2021). Characterization of DNA methylation and screening of epigenetic markers in polycystic ovary syndrome. Front Cell Dev Biol.

[37] Stelzer G (2016). The GeneCards suite: from Gene Data Mining to Disease Genome sequence analyses. Curr Protoc Bioinformatics.

[38] Song J (2019). Androgen upregulates NR4A1 via the TFAP2A and ETS signaling networks. Int J Biochem Cell Biol.

[39] Wang C (2016). Inhibition of melatonin metabolism in humans induced by chemical components from herbs and effective prediction of this risk using a computational model. Br J Pharmacol.

